# The Unintended Consequence of Novel Coronavirus (COVID-19) Pandemic on Racial Inequities Associated With Adverse Childhood Experiences (ACEs): Findings From a Population-Based Study

**DOI:** 10.3389/fpubh.2021.701887

**Published:** 2021-10-26

**Authors:** Aditi Srivastav, Chelsea L. Richard, Amanda S. McRell, Melissa Strompolis

**Affiliations:** ^1^Children's Trust of South Carolina, Columbia, SC, United States; ^2^University of South Carolina, Columbia, SC, United States; ^3^South Carolina First Steps, Columbia, SC, United States

**Keywords:** coronavirus, COVID-19, adverse childhood experiences, protective factors, structural racism

## Abstract

A rising concern is the COVID-19 pandemic effect on adverse childhood experiences (ACEs) due to increased parental stress and social/physical isolation. These pandemic effects are likely to be higher in already marginalized communities. The objective of this ecological study was to examine the relationship between COVID-19 cases and deaths, race/ethnicity, and the estimated number of adults with ACEs using data from South Carolina (SC). COVID-19 reported cases and death data were obtained from the SC Department of Health and Environmental Control. ACE data was used from the 2014–2016 SC Behavioral Risk Factor Surveillance System. Census data were used to obtain county population data. To measure the relationship between these variables, the Spearman rank-order correlation test was used because the data distribution was non-normal. There was a moderate relationship between the estimated number of adults with one or more ACEs and deaths (ρ = 0.89) and race/ethnicity-specific COVID-19 case counts by county (Black: ρ = 0.76; =White: ρ = 0.96; Hispanic: ρ = 0.89). Further, the Spearman correlation test showed the strongest relationship between COVID-19 deaths and race-ethnicity-specific county populations was with the Black adult population (ρ = 0.90). Given the known link between existing health inequities and exposure to COVID-19, these results demonstrate that the current pandemic could have unintended consequences on the well-being of children and caregivers. Response efforts should consider promoting protective factors for children and families and advocating for equitable policies and systems that serve children.

## Introduction

The COVID-19 pandemic has had devastating impacts on United States (US) populations. In addition to facing health impacts from the virus itself, increasing family stress and adversity related to the COVID-19 pandemic is negatively impacting children's health. Currently, children without underlying chronic health conditions are not deemed high risk for COVID-19 (1). Initial studies suggest that the illness presents milder symptoms in children compared to adults (1, 2). Despite this knowledge, there are growing concerns about the unintended consequences of COVID-19 on children's health and well-being as schools and afterschool activities remained closed, parents/caregivers navigate new stressors, and health care systems are stretched beyond capacity. For example, child welfare systems across the country have seen a reduction in child maltreatment rates and an increase in child abuse injuries (3). This may be due to children being socially and physically isolated from adults who are likely to be mandated reporters. Similarly, there are reports of increased domestic violence (4). This may be due to stay at home orders and the closure of community supports. Additionally, treatment and support systems for individuals with substance misuse issues may be disrupted which could lead to relapse or unsafe parenting behaviors within the home (5). Finally, due to lost wages associated with the closure of businesses, families that may have already faced financial hardship could be facing challenges in providing basic needs for their children which could lead to a stressful, unsafe environments.

These traumatic experiences are examples of Adverse Childhood Experiences, or ACEs, which have been extensively studied in relationship to several health and social outcomes in adulthood (6–9). Through the mechanism of toxic stress, ACEs impact the socio-emotional development of children which lead to poor health and social outcomes throughout a person's lifetime (10). Individuals with ACEs are more likely engage in risk behaviors, (11–13) report poor mental health and report poor physical health (11, 14) compared to individuals with no ACEs. Individuals with ACEs also have less access to health care (15). Parents/caregivers who experienced childhood trauma may have difficulties managing their own stress and teaching their children to manage stress (16, 17). In this way, parents/caregivers who experience ACEs and associated health difficulties may have children who also experience ACEs and associated health difficulties (18). Fortunately, the research on toxic stress has also shown the effects of traumatic experiences can be mitigated through the presence of protective factors, which include access to social supports, resources, and safe, stable nurturing relationships and environments (11, 14, 19). However, due to the current restrictions posed by COVID-19, both identified risks for ACE exposure and mitigating protective factors are likely to be detrimentally impacted by the pandemic (20).

Health consequences of structural racism further compound COVID-19-related stress and adversity. Overwhelming evidence establishes that structural racism is a root cause of inequities in public health (21). Research on ACEs has advanced a growing recognition of inequities embedded in exposure to adversity, namely through historical trauma and structural racism (15, 22–24). Child health scholars have called for an expansion of the definition of ACEs to include community and historical stressors that are likely to predispose children of color to these traumatic experiences (14, 23, 25). Preliminary data suggest that the causes and consequences of COVID-19 may mirror the causes and consequences of ACEs, as disproportionately more people of color have been exposed to and have died from the virus compared to their counterparts (26, 27). This is likely due to several systemic racial inequities, including people of color being more likely to work “essential” jobs that may be lower wage and lack adequate protection, having less access to high quality health care and rapid testing, as well as a lack of access to key resources such as stable housing, food security, or child care (26, 27). Taken together, the field of ACEs and the emerging knowledge on COVID-19 speak to the importance of viewing and addressing the effects of this pandemic beyond virus exposure and treatment, but recognizing, in our pandemic response, the impact the virus is having on key social determinants of health that influence health and well-being across the lifespan.

Thus, this ecological study examines the associations between COVID-19 rates (deaths and race/ethnicity-specific case counts; as of September 1, 2020) and prevalence of Adverse Childhood Experiences (ACEs) reported by adults using county level data from South Carolina. South Carolina has been one of the hardest hit states by the pandemic. As of September 20, 2021, there have been 159,108 cases per 1 million people in the state, which was the fifth highest in the US, compared to 127,642 cases per 1 million nationwide (28). Based on existing evidence, we hypothesize that counties with a high population of adults with one or more (1+) ACEs will also report high COVID-19 case and death counts compared to their counterparts. Given the role of structural racism in exposure to COVID-19, we predict that the relationship between high prevalence of ACEs and high prevalence of COVID-19 will be magnified in counties with more non-Hispanic Black adults.

## Materials and Methods

COVID-19 related data were obtained from the South Carolina Department of Health and Environmental Control (8, 29). ACE data was obtained from the 2014–2016 South Carolina Behavioral Risk Factor Surveillance System (29, 30). As was done in recent work by Brown et al. (31), this study employed a novel application of the BRFSS data, which are typically not powered for county level estimates. For the SC BRFSS however, regions are oversampled, so county level estimates can be obtained by aggregating three years of data for each county with at least 200 respondents.

The ACE questions included on the SC BRFSS mirrored those included in the original Felitti et al. (8). ACE study. These questions obtain information from non-incarcerated adults about their experiences before they were 18 years of age. County-level Census data from 2019 were obtained from the South Carolina Community Assessment Network. Institutional review was not needed as this study used publicly available, population level data.

The prevalence of at least one (1+) or more ACE were calculated by county (*n* = 46) using survey logistic procedures in SAS (32). These prevalence estimates were utilized to approximate the number of adults (18+) with each ACE exposure level by county using Census figures. To understand the normality of these data, the Shapiro-Wilk test was utilized because the sample size was small (*n* = 46). Because each Shapiro-Wilk test had a *p*-value equal to <0.001, each distribution was non-normal. Therefore, the Spearman rank-order correlation test was used to measure the relationship between these variables. Further, population counts were used instead of prevalence estimates because prevalence is bounded between 0 and 1; while, population counts are not.

## Results

In South Carolina, there are an estimated 4,030,737 adults (18+). As of September 1, 2020, there were 3,405 COVID-19 attributed deaths (75.5 deaths per 100,000 population). Further, there were 31,898 COVID-19 cases among Black adults (3,043.5 cases per 100,000 Black adults), 50,305 cases among White adults (1,870.2 cases per 100,000 White adults), and an estimated 12,360 cases among Hispanic adults (6,250.8 cases per 100,000 Hispanic adults). In South Carolina, 60.5% of adults have experienced one or more ACE. In SC, 65% of Black adults report one or more ACE, compared to 58% of their White counterparts. This is shown in Table 1.

**Table 1:** Statewide and county-level summary statistics (*n* = 46).

	**Mortality rate (COVID-19 deaths per 100,000 population)**	**Non-Hispanic Black incidence rate (COVID-19 cases per 100,000 Non-Hispanic Black adults)**	**Non-Hispanic white incidence rate (COVID-19 cases per 100,000 Non-Hispanic white adults)**	Hispanic incidence rate (COVID-19 cases per 100,000 Hispanic adults)*	**ACE incidence rate (%)**
Statewide	75.5	3,043.5	1,870.2	6,250.8	60.5%
Median	88.1	3,379.8	1,907.3	6,157.6	59.0%
Minimum	41.6	1,959.4	869.7	1,652.9	41.0%
Maximum	311.4	5,030.3	3,352.0	15,616.4	70.0%

COVID-19 data as of September 1, 2020; ^*^1 county had suppressed data due to low case count, so n = 45.

The Spearman correlation test showed there was a moderate relationship between the estimated number of adults with one or more ACEs and deaths (ρ = 0.89) and race/ethnicity-specific COVID-19 case counts by county (NHB: ρ = 0.76; NHW: ρ = 0.96; Hispanic: ρ = 0.89). Further, the Spearman correlation test showed the strongest relationship between COVID-19 deaths and race-ethnicity-specific county populations was with the Black adult population (ρ = 0.90). These results are further detailed in Table 2.

**Table 2:** Spearman rank correlation test results (*n* = 46; rho value (*p*-value) reported).

	**Estimated population of adults with ACEs**	**Population of Non-Hispanic white adults**	**Population of Non-Hispanic Black adults**	**Population of Hispanic adults**	**Total adult population**	**COVID-19 adult deaths**	**Non-Hispanic Black adult COVID-19 cases**	**Non-Hispanic white adult COVID-19 cases**	**Hispanic adult COVID-19 cases**
Estimated population of adults with ACEs									
Population of Non-Hispanic white adults	0.98 (<0.0001)								
Population of Non-Hispanic Black adults	0.85 (<0.0001)	0.77 (<0.0001)							
Population of Hispanic adults	0.93 (<0.0001)	0.92 (<0.0001)	0.74 (<0.0001)						
Total adult population	1.00 (<0.0001)	0.98 (<0.0001)	0.89 (<0.0001)	0.93 (<0.0001)					
COVID-19 adult deaths	0.89 (<0.0001)	0.84 (<0.0001)	0.90 (<0.0001)	0.79 (<0.0001)	0.90 (<0.0001)				
Non-Hispanic Black adult COVID cases	0.76 (<0.0001)	0.67 (<0.0001)	0.97 (<0.0001)	0.63 (<0.0001)	0.77 (<0.0001)	0.86 (<0.0001)			
Non-Hispanic white adult COVID cases	0.96 (<0.0001)	0.96 (<0.0001)	0.79 (<0.0001)	0.88 (<0.0001)	0.96 (<0.0001)	0.86 (<0.0001)	0.73 (<0.0001)		
Hispanic adult COVID-19 cases	0.89 (<0.0001)	0.88 (<0.0001)	0.69 (<0.0001)	0.95 (<0.0001)	0.88 (<0.0001)	0.73 (<0.0001)	0.59 (<0.0001)	0.85 (<0.0001)	

## Discussion

This ecological study shows that there is a potential relationship between reporting ACEs and COVID-19 cases and deaths, specifically for Black adults in South Carolina. Given the link between (1) structural racism and public health disparities, (2) COVID-19 and family stress, and (3) family stress and childhood adversity, these results suggest that the current pandemic may exacerbate the impact of ACEs for adults. Additionally, given the evidence of intergenerational trauma transmission (17) and the increased risk of ACEs for the children of adults who experience ACEs, (18) study results suggest that the stressors associated with the pandemic may create further exposure to ACEs for children, especially in predominantly Black communities in South Carolina, who have already reported higher rates of childhood trauma. Thus, this study underscores the importance of addressing the racial inequities that are being highlighted through this pandemic (27) to ensure that its effects do not increase exposure to childhood trauma.

COVID-19 response efforts should focus on promoting protective factors for children and families, especially children and families of color. This includes ensuring basic needs are met, including creating programs and policies that expand free testing for COVID-19, ensure stable housing by banning evictions and freezing utility bills during the pandemic, developing flexible unemployment benefits, expanding broadband access to remote and rural areas, adequate hazard pay and protection for essential workers, and ensuring food security through the expansion of community and state nutrition programs (12, 27, 33, 34). Additionally, to help build child and family resilience, response efforts should include parenting supports such as flexible work policies, accessible high-quality childcare, alternative educational options, and access to mental and emotional health supports. To maintain and help build positive child and family relationships, response efforts should promote and provide opportunities for positive childhood experiences including interactions between children and caring, supportive adults; engagement between children and safe, supportive communities; and participation for children in activities that develop social and emotional competencies (34). Finally, to dismantle the structural racism leading to disparate exposure to COVID-19, response efforts must call out and dismantle inherently unjust policies and systems contributing to the spread of the virus, including within health care access, law enforcement, criminal justice, housing, and employment (21, 35).

There are several well-known limitations to ecological studies, particularly the ecological fallacy. To explore this topic at the individual-level, data availability is a challenge. Right now, in most states, ACE prevalence is measured through state-added questions on the BRFSS, which is a cross-sectional survey among adults only, which can be subject to recall and social desirability biases, although estimates of ACEs in South Carolina are consistent with other statewide BRFSS surveys (36–38). The prevalence of ACEs in South Carolina is equivalent to that seen in 25 states (39); there is no national prevalence of ACE available to date. Measuring ACEs among children is difficult, given ethical considerations and mandated reporting requirements. The BRFSS has been widely accepted as the most rigorous way to understand ACEs on a population level; starting in 2021, the CDC will now include questions about ACEs in every state survey. Thus, the BRFSS data used in this study provides an important snapshot about the relationship between COVID-19 and adults who experienced childhood trauma, to help inform the way in which we can appropriately respond to the unintended consequences of the pandemic on children, especially given extensive literature studying ACEs among adults to understand its effects on children (40). Additionally, to date, race and other demographic data for COVID-19 cases and deaths are limited or delayed, with varying approaches to data collection and disaggregation across states. As of September 20, 2021, 9.5% of COVID-19 cases in SC and 5.0% of deaths were attributed to individuals with “unknown” race/ethnicity (41). As the pandemic rapidly evolves, our study remained limited to the data that are available, which are of varying quality. The varying quality of these data potentially underestimates the magnitude of the rho values of the current study, but most likely does not impact the direction of the values. Another limitation to the current study, based on data available, is the use of crude mortality rates by county, instead of age-standardized ones by race/ethnicity or gender. This is an important future direction of the current study. A final limitation of the current study is that the ACE data from the BRFSS is anonymous and, therefore, cannot be retroactively linked with COVID-19 case or death status. Future iterations of the BRFSS should consider asking about COVID-19 case history for a more rigorous study that employs data analysis at the individual-level for more robust population-level inferences about the association between ACE exposure and COVID-19 incidence.

The COVID-19 pandemic is providing us with another opportunity to address the underlying cause of public health disparities: structural racism. There have been several promising program and policy actions to help ensure communities are equipped to respond to the virus such as increased access to affordable testing in communities of color, access to parental resources through schools, and economic supports through state and federal stimulus efforts. In addition to the policy and program strategies aforementioned, on the other side of this pandemic, we should be adamant that these efforts continue, recognizing that child and family resilience should not only be defined by being able to overcome a traumatic experience but to be able to thrive despite experiencing adversity. COVID-19 has given public health an opportunity to take the reins and ensure that every child, every family, and every community can live a just and healthy life. In the aftermath of COVID-19 is, we must ensure that that optimal health and well-being is no longer determined by privilege (42).

## Data Availability Statement

Publicly available datasets were analyzed in this study. This data can be found here: https://scdhec.gov/covid19/covid-19-data/south-carolina-county-level-data-covid-19 and https://apps.dhec.sc.gov/Health/SCAN_BDP/tables/populationtable.aspx.

## Author Contributions

AS developed the overall research questions and led the research process. CR and AS designed the research and statistical analysis. MS provided guidance on the theoretical approach. AS, CR, AM, and MS wrote the paper and AS had responsibility for final content. All authors have read, contributed to and approved the final manuscript.

## Funding

This study was funded by the Blue Cross Blue Shield of South Carolina Foundation [Grant 2018-15].

## Conflict of Interest

The authors declare that the research was conducted in the absence of any commercial or financial relationships that could be construed as a potential conflict of interest.

## Publisher's Note

All claims expressed in this article are solely those of the authors and do not necessarily represent those of their affiliated organizations, or those of the publisher, the editors and the reviewers. Any product that may be evaluated in this article, or claim that may be made by its manufacturer, is not guaranteed or endorsed by the publisher.
